# Soil Moisture Levels Affect the Anatomy and Mechanical Properties of Basil Stems (*Ocimum basilicum* L.)

**DOI:** 10.3390/plants10071320

**Published:** 2021-06-28

**Authors:** Elisa Driesen, Maurice De Proft, Wouter Saeys

**Affiliations:** Department of Biosystems, KU Leuven, Willem de Croylaan 42, 3001 Leuven, Belgium; maurice.deproft@kuleuven.be (M.D.P.); wouter.saeys@kuleuven.be (W.S.)

**Keywords:** irrigation, mechanical properties, stem anatomy, flexural modulus, flexural strength, xylem, collenchyma, sclerenchyma

## Abstract

As plants would benefit from adjusting and optimizing their architecture to changing environmental stimuli, ensuring a strong and healthy plant, it was hypothesized that different soil moisture levels would affect xylem and collenchyma development in basil (*Ocimum basilicum* L. cv. Marian) stems. Four different irrigation set-points (20, 30, 40 and 50% VWC), corresponding respectively to pF values of 1.95, 1.65, 1.30 and 1.15, were applied. Basil plants grown near the theoretical wilting point (pF 2) had a higher xylem vessel frequency and lower mean vessel diameter, promoting water transport under drought conditions. Cultivation at low soil moisture also impacted the formation of collenchyma in the apical stem segments, providing mechanical and structural support to these fast-growing stems and vascular tissues. The proportion of collenchyma area was significantly lower for the pF1.15 treatment (9.25 ± 3.24%) compared to the pF1.95 and pF1.30 treatments (16.04 ± 1.83% and 13.28 ± 1.38%, respectively). Higher fractions of collenchyma resulted in a higher mechanical stem strength against bending. Additionally, tracheids acted as the major support tissues in the basal stem segments. These results confirm that the available soil moisture impacts mechanical stem strength and overall plant quality of basil plants by impacting xylem and collenchyma development during cultivation, ensuring sufficient mechanical support to the fast-growing stem and to the protection of the vascular tissues. To our knowledge, this study is the first to compare the mechanical and anatomical characteristics of plant stems cultivated at different soil moisture levels.

## 1. Introduction

The development and growth of land plants is highly dependent on water and nutrient transport throughout the plant. The xylem ensures the transport of water and minerals, powered by transpiration of the leaves. It is present in all vascular plants, with great variability across different plant groups and within plant species [1]. Understanding water transport, from the soil through the plant vascular system into the atmosphere, leads to innovations for optimization of plant breeding in the fields of drought resistance and adaptation to environmental changes [2]. Moreover, plants must constantly adapt their biomechanical performance during development under varying environmental conditions. This underlines the developmental plasticity rather than a genetically fixed design of the plant [3]. Due to global climate change, environmental conditions are expected to change. Predictions of future climate claim that fresh-water availability will decrease by 20% in Central Europe to 70% in the Iberian Peninsula [4,5]. These situations demand for the development of plants which are less sensitive to drought, and irrigation methods which obtain a higher water-use efficiency. Furthermore, irrigation management plays a crucial role in the economic and technical aspects of greenhouse-grown crops in containers. To prevent drought stress, water is typically provided in large quantities, resulting in over-irrigation and leaching out of minerals [6]. An optimal irrigation schedule could increase the water-use efficiency, while resulting in qualitative and quantitative crop improvements [7]. Increased insights in the influence of soil moisture on vascular development, especially that of xylem vessels, can lead to new approaches for adaptation of plants and irrigation management to climate change.

In vascular plants, the xylem has two main functions: (i) transporting water to all sink tissues, and (ii) providing mechanical support [2]. Xylem vessels of angiosperms are non-living cells with wide lumens and lignified cell walls, specialized to conduct water, stacked end-to-end for maximal efficiency [8]. Xylem tracheids are elongated narrow-tube cells with thick lignified cell walls, conducting water and minerals, and providing mechanical support. Lignin contributes to the mechanical strength of xylem vessels and tracheids through its rigidity [9]. Weak lignification of primary cell walls reduces cell adhesion among tracheids and xylem vessels, thus reducing their capacity to withstand mechanical stresses associated with water transport [9]. Several researchers have reported the relationship between xylem anatomy, drought tolerance and water-use efficiency [8,10]. However, little research has been published on the possible trade-offs between stem mechanical strength and water-transport efficiency. This relationship is determined by the cylindrical shape of the stem and the physical properties of the xylem vessels present. An increase in stem diameter will impact its stiffness and thereby its mechanical strength [11]. Vessel diameter and frequency are factors determining the water-flow efficiency through the conduits [2]. Drought stress has been reported to reduce vessel diameter and thickness of vascular cambium, and to delay the formation of the xylem and phloem in woody plants [5]. 

Stem mechanical strength is not only determined by vascular tissues (xylem vessels and tracheids), but also by collenchyma (composed of living cells) and sclerenchyma (composed of dead cells), which are crucial for mechanical support in angiosperms [12,13]. Collenchyma provides mechanical support to rapidly growing organs thanks to its low compressibility and relatively high moduli of elasticity [11]. In herbaceous plants, collenchyma is located immediately under the stem epidermis as peripheral collenchyma. The cell walls of collenchyma are made up of cellulose microfibrils, which are embedded in a hydrated matrix of hemicelluloses and pectins, forming a strong network [14,15]. Collenchyma as well as sclerenchyma serve as the main supporting tissues in herbaceous stems, with collenchyma having an important mechanical role in stem growth and development. Sclerenchyma provides rigidity as well as tensile and shear strength to many plant organs. However, it is incapable of supporting growing plant organs because it is composed of dead cells with non-extensible rigid cell walls [15]. While collenchyma typically occur in the peripheral regions of growing stems, sclerenchyma are only formed when the elongation has ceased [16]. Although the above-mentioned definitions are widely accepted, the distinction between both tissues is not always clear cut. Collenchyma may undergo lignification, while some sclerenchyma tissues develop non-lignified pectin-rich cell walls, resembling collenchyma. To avoid confusion, sclerenchyma will be used to describe tissues with rigid, lignified cell walls [16]. Several researchers have already investigated their relative contributions to the mechanical properties of a stem, supporting the hypothesis that collenchyma and sclerenchyma are the main supporting tissues protecting the stem against tension and bending loads [17,18,19].

Ambient temperature, precipitation and overall soil moisture were reported to contribute to significant changes in strength of stem wood [20]. Lower soil moisture content is correlated with stronger and denser wood [20,21,22]. Stem strength, like stem wood, may also be higher when exposure to drier soil conditions was experienced during plant development, increasing cellulose and lignin content. However, the influence of soil moisture on the development of these tissues, crucial for mechanical support of stems, is not described in the literature. The possibility that stem strength may differ due to differences in soil water content is investigated in this article, focusing on the influence of water availability on xylem, collenchyma and sclerenchyma formation, and its relation to mechanical stem strength. Additionally, changes in xylem development and structure determine the water use of plants and therefore the overall water management. Xylem cells form as a result of cell production and differentiation occurring in the cambium, a process that is influenced by both endogenous and environmental factors. Differentiation follows cell division [23] and determines the specific anatomical characteristics of the xylem. Xylogenesis, the process of conduit formation through programmed cell death, involves non-living cells, thereby making the timing of xylogenesis important [5].

To pave the way towards precision irrigation, we evaluated the effects of irrigation control based on different substrate volumetric water content (VWC) thresholds on the development of the xylem, collenchyma and sclerenchyma, in addition to their anatomical traits. Their anatomical traits in the basil stems, both apical as basal, were measured to determine which structural traits may be associated with the substrate water availability and stem mechanical strength. The experiments were conducted with basil (*Ocimum basilicum* L. cv. Marian), which is a greenhouse crop common in the Mediterranean diet, utilized to enhance food flavor. Our objectives were: (1) to study the effects of soil moisture thresholds on xylem, collenchyma and sclerenchyma formation in basil stems; and (2) to assess the contribution of collenchyma, sclerenchyma and xylem vessels and tracheids to the bending strength of the stems.

## 2. Results

### 2.1. Substrate Matric Potential during Cultivation

According to the substrate-specific water retention curve, the pF values (log_10_ of the substrate matric potential in hPa) at average substrate VWC of 20%, 30%, 40% and 50% were 1.95, 1.65, 1.30 and 1.15, respectively. These values correspond to matric potentials of −89, −45, −20 and −14 hPa. Plants from treatment ‘pF1.95’ were cultivated near the theoretical wilting point (−100 hPa), while the plants from treatment ‘pF1.15’ were cultivated near container capacity (−10 hPa). During container cultivation the water in the substrate is generally considered available for plants between matric potentials of −10 to −100 hPa [6]. The water availability in the drought-stressed treatment (‘pF1.95’) in our experiment could not be considered a limiting factor for plant growth, as the total dry weight of stems and leaves was not significantly reduced compared to the other treatments. The dry weight of stems and leaves for treatments ‘pF1.95’, ‘pF1.65’, ‘pF1.30’ and ‘pF1.15’ was 3.44 ± 0.68, 3.51 ± 0.48, 3.75 ± 0.27 and 3.78 ± 0.58 g container^−1^, respectively. Plant containers from treatments ‘pF1.30’ and ‘pF1.15’, did show significant higher evapotranspiration values, 1180 ± 303 mL container^−1^ and 1054 ± 285 mL container^−1^ respectively, compared to the drier treatments, with values of 769 ± 86 mL container^−1^ and 797 ± 8 mL container^−1^ for ‘pF1.95’ and ‘pF1.65’, respectively. As a result, the water-use efficiency (WUE) (g dry matter L^−1^) was significantly higher for treatments ‘pF1.95’ and ‘pF1.65’, with values 2.5 ± 0.2 and 2.4 ± 0.3 g dry matter L^−1^, compared to treatments ‘pF1.30’ and ‘pF1.15’, with values 1.5 ± 0.2 and 1.7 ± 0.3 g dry matter L^−1^, respectively. 

### 2.2. Xylem Anatomical Traits 

**Figure 1:**
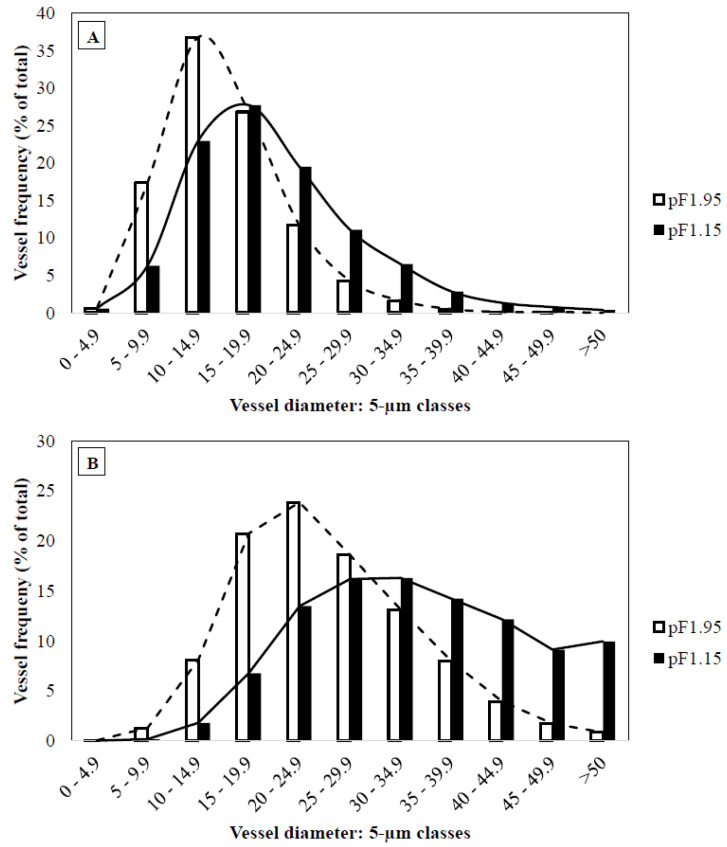
Frequency distribution of the 5 µm vessel diameter classes for treatments ‘pF1.95’ and ‘pF1.15’ in apical (**A**) and basal (**B**) stem segments.

Transverse sections of basil internodes showed a typical dicotyledonous stem structure. Vessel diameter frequency in 5 µm diameter classes is presented together with their relative contribution to each diameter class (Figure 1). The distribution of vessel diameters varied with distance from the roots. In apical stem segments, vessel diameter was generally smaller compared to vessels from the basal stem segments for all treatments. The modal diameter class was 10–14.9 µm for treatment ‘pF1.95’ and 15–19.9 µm for treatments ‘pF1.65’, ‘pF1.30’ and ‘pF1.15’ for the apical stem segments, while the modal diameter class for the basal stem segments was 20–24.9 µm (‘pF1.95’) and 25–29.9 µm (‘pF1.65’, ‘pF1.30’ and ‘pF1.15’). The frequency distributions of the vessel diameter classes for treatments ‘pF1.65’ and ‘pF1.30’ were similar to those for the ‘pF1.15’ treatment, both for the apical and basal stem segments (data not shown). 

The frequency distribution of apical stem segments from treatment ‘pF1.95’ was skewed to the left, with many small vessels and few large vessels (Figure 1A). Vessel diameter frequency distribution approaches a unimodal pattern, with a peak at 10–14.9 µm for ‘pF1.95’ and a peak at 15–19.9 µm for ‘pF1.15’ in the apical stem segments. More than half of the vessels from plants in treatment ‘pF 1.95’ (54.8%) had a diameter smaller than 15 µm, and only 2.3% showed a vessel diameter larger than 30 µm. The 10–14.9 µm interval class had the highest vessel frequency, 36.8%. Contrarily, 11.9% of the vessels from plants in treatment ‘pF1.15’ showed a vessel diameter larger than 30 µm, while 27.7% of the vessels had a diameter ranging between 15 and 19.9 µm (Figure 1A). The frequency of vessels with diameters larger than 15 µm was higher for treatments ‘pF1.65’, ‘pF1.30’ and ‘pF1.15’ compared to ‘pF1.95’. Similar results, but higher values, were obtained for the frequency distribution of the basal stem segments (Figure 1B).

Xylem anatomical traits in the drought treatment ‘pF1.95’ show adjustment to drought conditions by increasing the vessel frequency per mm^2^ and decreasing the mean vessel diameter (Table 1). Lower soil moisture content during the cultivation period resulted in significantly smaller xylem vessels, with a mean diameter of 15.42 ± 1.62 µm (apical) and 25.29 ± 2.39 µm (basal). Although vessel frequency per mm^2^ in the apical segments was significantly increased for the ‘pF1.95’ treatment, with 83 ± 17 vessels per mm^2^ compared to ‘pF1.65’ (49 ± 7 vessels per mm^2^), ‘pF1.30’ (55 ± 11 vessels per mm^2^) and ‘pF1.15’ (48 ± 12 vessels per mm^2^), the total percentage vessel area per transverse stem area [%] was not significantly different between treatments. The percentage total vessel area per transverse stem area did significantly differ between treatments for the basal stem segments, with stems from treatment ‘pF1.65’ having the significant highest percentage (2.57 ± 0.43%) and stems from treatment ‘pF1.95’ the lowest percentage (1.99 ± 0.43%). The mean vessel diameters did not significantly differ between treatments ‘pF1.65’, ‘pF1.30’ and ‘pF1.15’, both for the apical and basal stem segments. 

**Figure 2:**
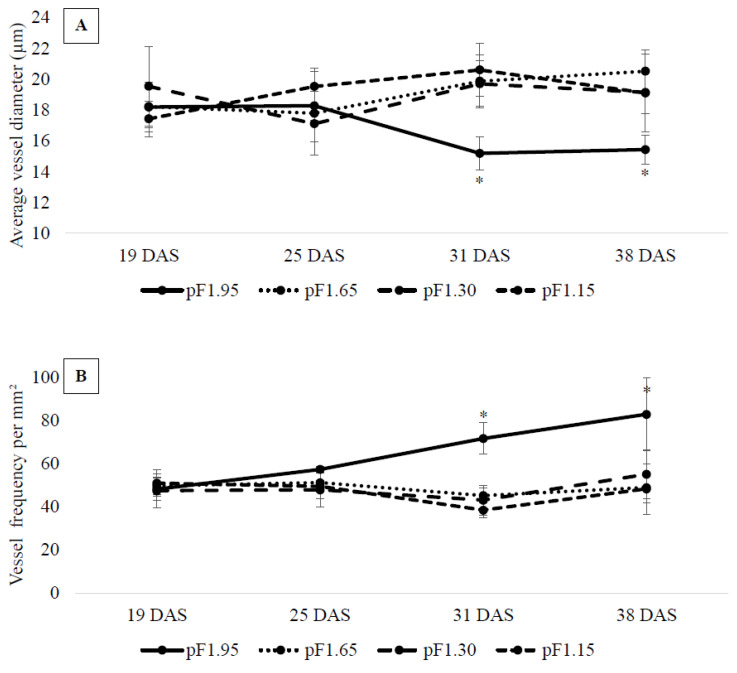
Average vessel diameter (µm) (**A**) and vessel frequency per mm^2^ (**B**) for apical segments of basil stems for treatments ‘pF1.95’ (solid line), ‘pF1.65’ (dotted line), ‘pF1.30’ (wide dashed line) and ‘pF1.15’ (short dashed line) during the cultivation period, at 19, 25, 31 and 38 DAS. An asterisk indicates a significant difference (*p* < 0.05) between the different treatments at those DAS.

This significant difference in average vessel diameter and vessel frequency per mm^2^ of apical stems from treatment ‘pF1.95’ manifested itself early in the cultivation period (Figure 2). While the vessel frequency for plants of treatments ‘pF1.65’, ‘pF1.30’ and ‘pF1.15’ did not significantly differ during the cultivation period, the vessel frequency for treatment ‘pF1.95’ increased during the cultivation period starting from 25 DAS (days after sowing). It was significantly different from the other treatments at 31 and 38 DAS, with 71 and 83 vessels per mm^2^, respectively (Figure 2B). Similarly, the average vessel diameter decreased during the cultivation period for the drought treatment, with significant differences at 31 and 38 DAS compared to the other treatments (Figure 2A). 

### 2.3. Collenchyma Anatomical Traits and Mechanical Strength of Apical Stem Segments 

**Figure 3:**
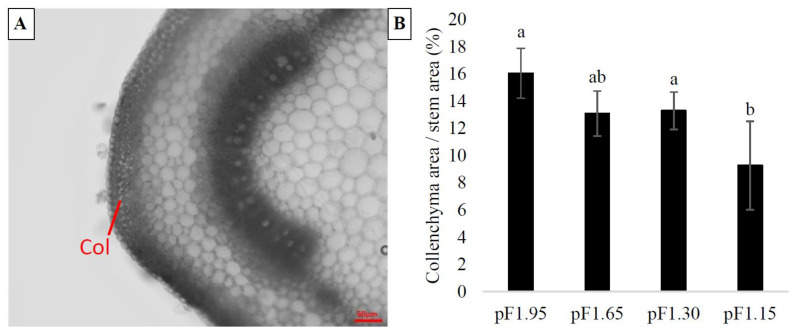
(**A**): micrograph of a transverse section of an apical stem segment, illustrating the location of collenchyma cells (two to three layers) underneath the epidermal layer. The scale bar represents 50 µm. (**B**): percentage total collenchyma area per stem area ± SD (*n* = 20) for the different irrigation treatments. Different letters indicate significant differences between the treatments according to a Tukey test (*p* ≤ 0.05).

Collenchyma tissue can be recognized by its unevenly thickened cell walls with high cellulose content. In the apical stem segments, collenchyma is located in a peripheral position, immediately beneath the epidermis (Figure 3A). The collenchyma cells are located angularly, associated with the vascular bundles. The type of collenchyma in our basil stems is angular collenchyma, which is recognizable by the heavily thickened cell walls occurring at the angles of the cell surface where several collenchyma cells converge [15]. 

The percentage of stem area which was occupied by collenchyma showed a significant decrease at higher soil moisture content for the apical stem segments (Figure 3B). Plants cultivated by treatment ‘pF1.95’ showed 73% more collenchyma area per stem area compared to the ‘pF1.15’ treatment. The collenchyma area per stem area was increased respectively by 41% and 44% for treatments ‘pF1.65’ and ‘pF1.30’ compared to the container-capacity treatment (‘pF1.15’). In contrast to xylem vessel area, collenchyma occupied a larger percentage of the stem area of the apical stem segments, 16.04%, 13.08%, 13.28% and 9.25% for the ‘pF1.95’, ‘pF1.65’, ‘pF1.30’ and ‘pF1.15’ treatments, respectively, while xylem vessel area occupied 1.25%, 1.39%, 1.29% and 1.11%, respectively. The collenchyma cells did not differ significantly in size, as the mean collenchyma diameters of the different treatments were not significantly different, with 11.20 ± 4.13 µm, 11.83 ± 4.53 µm, 12.18 ± 4.89 µm and 12.83 ± 4.70 µm being the average collenchyma cell diameters for treatments ‘pF1.95’, ‘pF1.65’, ‘pF1.30’ and ‘pF1.15’, respectively. 

Mechanical bending tests were conducted using different stems from the same plant containers used for the microscopical measurements. The Wilcoxon test of the data indicated that the soil moisture content during cultivation had a significant effect on the resistance in bending of the plant stems (*p* ≤ 0.05). The flexural modulus (*E_bend_*) and flexural strength (*σ*) of treatments ‘pF1.95’ and ‘pF1.65’ were significantly higher compared to treatment ‘pF1.15’. The flexural modulus and strength decreased with an increasing soil moisture content, indicating that the stems become less stiff (Table 2). 

**Figure 4:**
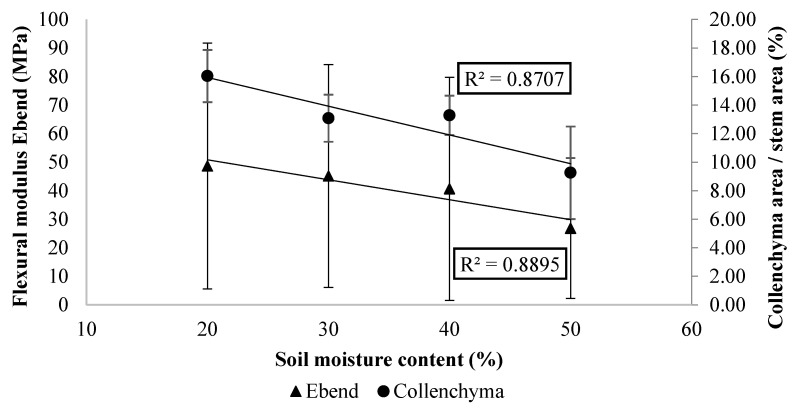
Relationship between the soil moisture content (%), the flexural modulus Ebend (MPa) and the percentage collenchyma area per stem area.

This reduction in the modulus of elasticity of the apical stem segments with increasing soil moisture is correlated with the collenchyma area per stem area (Figure 4). Stems with a higher resistance against bending, cultivated at lower soil moisture contents, are characterized by a greater contribution of collenchyma to the overall stem cross section. However, these stems have a similar proportion of xylem area. These results are consistent with the hypothesis that collenchyma cells are the most responsible for resistance to bending in these apical stem segments. 

### 2.4. Tracheid and Sclerenchyma Anatomical Traits and Mechanical Strength of Basal Stem Segments 

**Figure 5:**
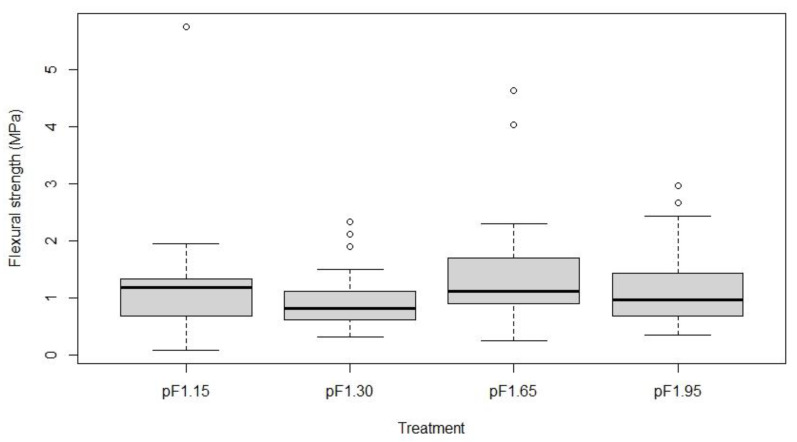
Box and whisker plot of the flexural strengths (*σ*) (MPa) of the basal stem segments from different irrigation levels (*n* = 30) during the cultivation period.

The basal stem segments showed sclerenchyma formation as well as secondary xylem, containing vessels and tracheids. Tracheid mean diameters and area were also measured for the basal stem segments, as they are also important for mechanical strength. The basal stem segments for the four different irrigation treatments displayed no significant differences in the proportion of tracheid and sclerenchyma areas per stem area, nor in the mean diameter of the cells (Table 3). This suggests that water availability during the cultivation period had no significant effect on tracheid and sclerenchyma formation in the basal stem segments (*p* > 0.05). Additionally, the basal stem segments of different irrigation levels did not differ in terms of flexural strength and flexural modulus (Figure 5). 

**Figure 6:**
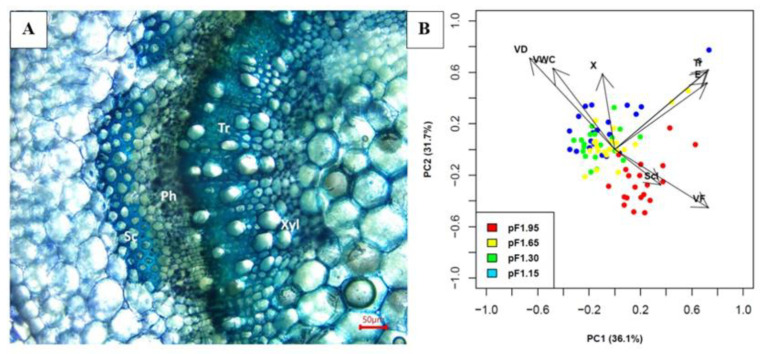
(**A**): anatomy of basal stem segments at 20x magnification, stained with Toluidine Blue. The scale bar represents 50 µm. Abbreviations: Sc, sclerenchyma; Ph, phloem; Xyl, xylem; Tr, tracheids. (**B**): Principal component analysis (PCA) biplot showing the different treatments with ‘pF1.95 ‘(red), ‘pF1.65’ (yellow), ‘pF1.30’ (green) and ‘pF1.15’ (blue). This analysis combines all stem anatomical traits (VF, VD, X, Scl, Tr) as well as the mechanical traits (E and s) for the basal stem segments of the four different treatments. Abbreviations: VD, vessel diameter; VWC, substrate volumetric water content; X, xylem area per stem area; Scl, Sclerenchyma area per stem area; s, flexural strength; E, flexural modulus; VF, vessel frequency and Tr, tracheids.

The cell walls of xylem vessels, tracheids and sclerenchyma were lignified as evidenced by staining with Toluidine Blue (Figure 6A). To gain insights into the relative importance of the vascular tissues (xylem vessels and tracheids) and the mechanical tissue sclerenchyma on the basal mechanical stem stiffness, a principal component analysis was conducted between the stem’s structural morphology and mechanical properties, depending on the water availability during the cultivation period. The PCA included the following parameters: substrate volumetric water content (VWC), vessel diameter (VD), relative vessel area (X), vessel frequency (VF), *E_bend_*, *σ*, sclerenchyma area (S) and tracheid area (Tr). The principal component analysis clearly separated VWC from mechanical stem strength, both *E_bend_* and *σ*, as shown by the 90° angle of these vectors on the biplot. The first component explained 36.1% of the variation in the dataset and showed strong positive loadings with tracheid area (0.72), *E_bend_* (0.72), *σ* (0.73) and vessel frequency (0.73), and strong negative loadings with vessel diameter (−0.66). The first component gives evidence for an association between xylem formation and mechanical stem strength against bending. The second principal component explained 31.7% of the variation in the dataset and is strongly positively correlated with the VWC (0.63), vessel diameter (0.71) and relative vessel area (0.59), and negatively correlated with vessel frequency (−0.45). This second principal component can therefore be interpreted as the trade-off between xylem anatomical traits and water availability during the cultivation period, as it is evident that this PC clustered all parameters associated with the xylem vessels and no parameters associated with sclerenchyma. The stems from the drought treatment (‘pF1.95’) exhibited high vessel frequency with small diameters, while the non-drought treatments (‘pF1.65’, ‘pF1.30’ and ‘pF1.15’) showed vessels with a larger diameter at low frequency. Sclerenchyma area was loaded with PC 3 (0.57), explaining 14.2% of the variation in the dataset, with relative vessel area (−0.65) and treatment (0.44).

There was a strong correlation (*p* ≤ 0.01) among the tracheid area and mechanical strength parameters, with correlation values of 0.76 and 0.86 for *E_bend_* and σ respectively (Table 4). Tracheids provided the main structural support of basil stems at the base of the stem, while sclerenchyma area had no significant correlation with the mechanical strength parameters. A weak correlation between vessel frequency per mm^2^ and flexural strength (0.21), and vessel area and flexural strength (0.27) was also observed. 

**Figure 7:**
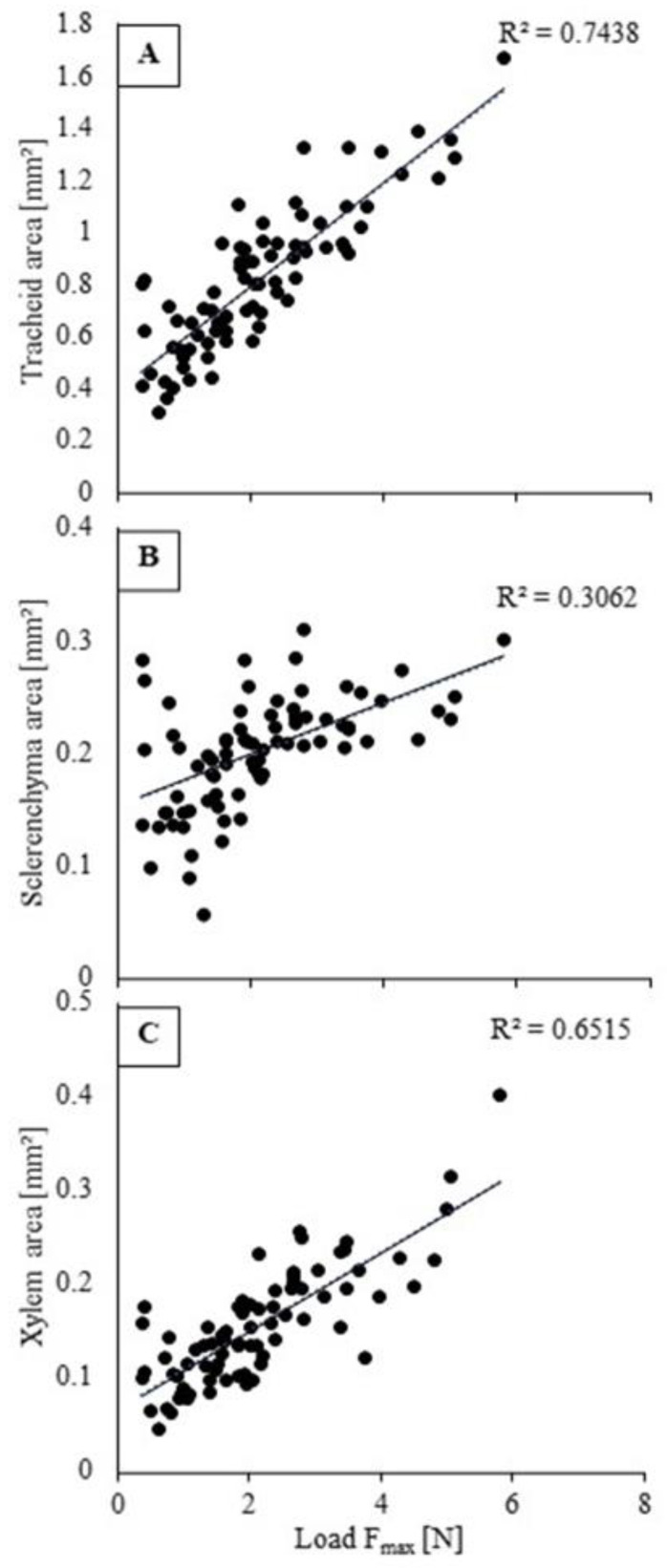
Linear correlations between stem structural properties and the maximal load (*F_max_*) (N): (**A**) tracheid area (mm^2^); (**B**) sclerenchyma area (mm^2^); and (**C**) xylem area (mm^2^).

A stem’s maximal resistance to bending force (*F_max_*) is directly proportional to the tracheid area (mm^2^) (R^2^ = 0.74) and the total xylem area (mm^2^) of that stem area (R^2^ = 0.65) (Figure 7). A weaker relationship was found between the sclerenchyma area (mm^2^) and the bending force (*F_max_*) (R^2^ = 0.31). Plant stems with a higher number of tracheids and total xylem area display a higher maximal load (*F_max_*), translating to higher forces necessary to break the stem sample. This shows that xylem vessel formation gives additional strength to the stems, which explains the correlation of vessel area per stem area (0.27) and vessel frequency per mm^2^ (0.21) with flexural strength (*σ*). The proportion of tracheid area per stem area was 89%, 86%, 86% and 88% higher for treatments ‘pF1.95’, ‘pF1.65’, ‘pF1.30’ and ‘pF1.15’, respectively, when compared to the proportion of xylem area per stem area. Similarly, sclerenchyma took up a smaller proportion of the total stem area compared to tracheids, 80%, 82%, 79% and 82%, respectively. 

## 3. Discussion

### 3.1. Soil Water Availability Influences Xylem Anatomical Traits

The obtained results illustrate the adaptive relationship between soil water availability and xylem vessel diameter and frequency. The higher water stress in the ‘pF1.95’ treatment imposes safety constraints, reducing vessel diameter whilst increasing vessel frequency. This way, plants ensure their water conductivity with narrower and safer conduits where water potentials are more negative [24]. Similar results were reported by February (1993) working on *Combretum apiculatum* and *Protea caffra*, where xylem vessel size decreased with intensifying aridity, while vessel frequency per mm^2^ increased [25]. In line with our results, smaller xylem diameters were reported in response to drought in poplar trunks (*Populus nigra* L. × *Prunus maximowiczii*) [26]. Large and long vessels are also more prone to cavitations, which are caused by the formation of air emboli in conduits, interrupting upward water movement [27]. Weak pit structures and large xylem diameters increase the vulnerability to cavitations [28,29]. According to the law of Hagen–Poiseuille, water conductivity corresponds to the fourth power of the conduit diameter [29]. Consequently, hydraulic conductivity can be considerably increased by increasing the xylem diameter. Interestingly, the plants exposed to drier conditions (‘pF1.95’ and ‘pF1.65’) displayed a higher WUE during the cultivation period. Plant containers from treatments ‘pF1.30’ and ‘pF1.15’ did show significant higher evapotranspiration values, 1180 ± 303 mL container^−1^ and 1054 ± 285 mL container^−1^, respectively, compared to the drier treatments, with values of 769 ± 86 mL container^−1^ and 797 ± 8 mL container^−1^ for ‘pF1.95’ and ‘pF1.65’, respectively. As the dry weight of leaves and stems did not differ between treatments, the WUE, expressed as (g dry matter L^−1^), did significantly differ. Smith et al. (2013) found an indication of higher resistance to cavitation in species with a higher number of vessels per xylem area than those with less vessel area per xylem area when examining the hydraulic properties of 82 native and non-native woody species common to forests in Eastern North America [30]. In our study, this adaptive relationship between soil water availability and xylem frequency was confirmed for basal stem segments in basil. Traits associated with the xylem (vessel diameter and area) were positively influenced by the substrate volumetric water content during the cultivation cycle, with high loading values indicating that the positive association was strong. A strong negative correlation between substrate volumetric water content and vessel frequency (r^2^ = −0.68) and a strong positive correlation between substrate volumetric water content and vessel diameter (r^2^ = 0.67) was found (Table 4).

We found that irrigation management influences xylem cell differentiation early in the developmental stage of basil plants (Figure 2). At 31 DAS, the average vessel diameter and the vessel frequency per mm^2^ of the apical stem segments from the drought treatment showed significant differences compared to the non-drought treatments. Vessel frequency increased by 69% (31 DAS) and 63% (38 DAS), while vessel diameter decreased significantly by 24% (31 DAS) and 20% (38 DAS) compared to the pooled non-drought treatments. By adjusting and optimizing its hydraulic architecture to environmental stimuli, the plant will benefit from this developmental adaptation. Xylem formation, or xylogenesis, is a result of cambium activity, which is regulated by endogenous factors as well as environmental stimuli. Being a dynamic process, controlled by environmental conditions, the anatomy of the xylem reflects the water availability during the cultivation period. Persistent drought periods can directly and indirectly influence cell differentiation [29]. On the one hand, the rate of cell division and differentiation is influenced by water availability, reflecting the role of water availability in vessel ontogeny [31]. Water availability directly influences the xylem formation in the time window during which the cells are developing. During development, the cambial derivatives will alter both morphologically and physiologically, as explained by Rossi, Deslauriers and Anfodillo (2006) for wood production. In essence, derivatives consist of a protoplast enclosed in the primary cell wall, enlarging by water movement into the vacuole as a result of a water potential gradient across the cell wall [32]. Cell enlargement arises due to loosening of the cell wall, which lowers the turgor pressure. This is followed by an osmotically generated influx of water which increases the turgor pressure and results in an expanding cell wall [32]. This positive turgor pressure depends on the uptake of water provided during cultivation, resulting in an increase in radial diameter of the vessel during high water availability. Once their final size has been reached, the cells will mature through cell wall thickening and lignification, forming xylem vessels [23,31]. On the other hand, persistent drought periods can also indirectly induce developmental changes by influencing the availability of photoassimilates [29]. 

### 3.2. Collenchyma and Sclerenchyma Formation Changes with Soil Water Availability

The VWC during the cultivation period influenced the collenchyma formation, resulting in a difference in the mechanical strength of the stems. The percentage of collenchyma area per stem area showed a significant decrease at higher soil moisture content, with 73% more collenchyma area in apical stems segments for the drought treatment (‘pF1.95’) compared to the container-capacity treatment (‘pF1.15’). The difference in collenchyma formation cannot have been turgor driven, as the mean diameters of collenchyma cells did not significantly differ between the irrigation treatments. This confirms that the higher collenchyma area in plant stems exposed to lower soil moisture contents is the result of the formation of additional collenchyma cells. Considering the stem segments were brought to full turgidity before performing the mechanical bending test, and that stem segments of comparable size were used, this difference in stem strength can be attributed to collenchyma formation and not to the loss of turgor in these cells [13]. The flexural strength (*σ*) and the flexural modulus (*E_bend_*) decreased with an increase in the soil moisture content, which indicates a reduction in the stiffness of the stems. Locating strong tissues towards the outer edge of the stem is advantageous for their overall bending strength, as bending stresses under a load increase away from the central axis [19]. In contrast, irrigation does not seem to have an influence on sclerenchyma formation found at the basal stem segments. Water availability during the cultivation period had no significant effect on sclerenchyma formation, as the sclerenchyma area was similar between treatments. Plant cells have been reported to dedifferentiate and regenerate tissues under certain conditions, referred to as totipotency [33]. There are exceptions for certain types of terminally differentiated cells, i.e., highly lignified cells such as mature fibers and sclereids. Somatic cells, such as parenchyma, collenchyma and mesophyll cells can be reprogrammed as a reaction to internal (developmental) or external (stress) signals. This will result in dedifferentiation to become competent for switching fate, which can occur during the normal developmental program of plants [33,34]. This could explain the increase in collenchyma cells in the apical stem segments during drought stress (Figure 3). Modification of the stem structure under drought stress could be an adaptation to prevent cellular collapse. Olmos et al. (2006) studied the modification of the structure of *Rosmarinus officinalis* leaves under long-term drought stress and found a reduction in cellular size of the adaxial epidermal cells, increased cell-wall size and lignification of collenchyma and sclerenchyma. As basil is a fast-growing plant, turgor pressure may not provide sufficient support for the fast-growing organs, especially under drought conditions. Additional support for these young tissues is subsequently provided by collenchyma with its thick, flexible cell walls and protoplasts capable of resuming biological activity [15,35].

### 3.3. Stem Anatomical Structures Contribute to its Mechanical Strength against Bending

The formation of xylem vessels could also contribute to mechanical stem strength, as the apical stem segments from the drought treatment displayed a higher vessel frequency per mm^2^, hypothetically increasing the lignin content with a larger number of smaller vessels. In contrast, collenchyma cell area per stem area in the apical stem segments were quite different from the xylem vessel area per stem area. For example, 16.04% of the total collenchyma was present underneath the epidermis of the apical stem segments in the drought treatment, while the total area of xylem in the apical stem segments in the drought treatment was 1.25%. Esau (1936) compared the strength of collenchyma and vascular bundles of celery petioles by determining the breaking load of individual strands of these tissues. It was found that collenchyma strands were much stronger than xylem vessels, as the breaking load of collenchyma was two to four times that of the vascular bundles [17]. It is apparent that collenchyma contributes the most to mechanical stem strength against bending in the apical stem segments. 

The first irrigation experiment was replicated to find a statistical correlation between the anatomical characteristics of the stem segments and mechanical stem strength against bending, because in the first replication the anatomical traits were determined on different stems than those used for the mechanical bending tests, so a PCA and correlation analysis could not be performed. However, collenchyma cells were not present in the basal stem segments. As collenchyma typically occurs in the growing regions of herbaceous stems and petioles, it was prominent in the apical stem segments and absent in the basal stem segments [15,16,17] where secondary growth occurred. Instead, sclerenchyma is supposed to offer rigidity to the plant stem at the stem base. Tracheids surrounding the xylem vessels were prominent in the basal stem segments, theoretically offering both conductive and supportive functions within the secondary xylem [36]. The flexural modulus and flexural strength decreased towards the upper regions of the stem. Flexural strength was two to three times higher in the basal stem segments compared to the apical stem segments (Table 2, Figure 5). This may be explained by the combination of larger stem diameters (22 to 43% larger) and different mechanical tissues in basal stems, such as sclerenchyma and tracheids, compared to collenchyma in the apical stem segments. 

The correlations between xylem and sclerenchyma anatomical traits and mechanical characteristics (flexural modulus and strength) yield further insights into the contribution of xylem and sclerenchyma in mechanical stem strength against bending. A PCA was used to examine the relationship among the basal stem traits simultaneously. For the basal stem segments, traits associated with mechanical strength (*E_bend_* and *σ*) clustered with the proportion of tracheid area and loaded strongly along PC 1 (Figure 6). Additionally, vessel frequency and vessel diameter also loaded strongly along PC 1. A correlation analysis also showed a strong correlation among tracheid area and *E_bend_*, and *σ*, of 0.76 and 0.86, respectively. While tracheid area showed a strong correlation with mechanical strength parameters, sclerenchyma area had no significant correlation with these parameters. Furthermore, a weak correlation was found between vessel frequency and area, and flexural strength, of 0.21 and 0.27, respectively. Further analysis of the maximal resistance to bending load (*F_max_*) showed a linear relationship with both tracheid area and total xylem area (Figure 7). As the proportion of tracheid area was higher compared to the proportion of xylem area per stem area and sclerenchyma area, this could explain the strong correlation between mechanical strength parameters and tracheid area compared to the weaker correlation with sclerenchyma area. Furthermore, the mean diameters of tracheids are considerably smaller than the xylem vessels, up to 69% smaller for the container-capacity treatment and 58% smaller for the drought treatment. Tracheid dimensions may be more limited as they have the dual function of conductivity and mechanical support, facing a possible trade-off between these functions [8,36,37]. Safe xylem transport is characterized by resistance to xylem implosion, resulting in a fiber matrix surrounding the vessels to protect them from collapsing under negative pressures [21,38,39]. This is consistent with our findings, with tracheids acting as the major support tissue when bending stresses are imposed on the basal stem segments. Xylem safety, such as increasing xylem frequency and decreasing vessel diameters from cavitation, is therefore positively correlated with more robust tissues [40].

## 4. Materials and Methods

### 4.1. Plant Material and Growth Conditions 

Basil *(Ocimum basilicum* L. cv. Marian) seeds were planted in soil containing peat, compost and perlite, and grown in biodegradable containers (D-grade Bio^®^) with a total volume of 230 cm³ and an average of 15 ± 4 plants per container. After sowing, the containers were kept in a germination chamber for 2 days at 90% relative humidity, with a temperature of 21 °C in total darkness. Containers were transferred to the growth chambers in trays (7 containers × 5 containers) after two days (4 DAS). Each growth chamber (2 m^2^) contained 2 trays, totaling 70 containers. The climate in the growth chambers was controlled to obtain a 16 h photoperiod, 24 °C/19 °C temperature, 180 µmol m^−2^ s^−1^ light intensity and 70% relative humidity. A broad white-light spectrum was implemented [41], using LED multispectral lamps suspended at a height of 112 cm above the plants. The light intensity was lowered, following the plant growth, keeping 180 µmol m^−2^ s^−1^ light intensity at plant level. A 16-h photoperiod was set from 6:00 a.m. to 10:00 p.m. Irrigation was implemented using a flood and drain system, with a water level of 3.1 ± 0.1 cm at maximum height and an irrigation time of 4 min. The nutrient solution (NS) was kept at an electrical conductivity (EC) of ≈ 0.7 mS/cm at 21 °C. The macronutrient concentration of the NS was: 4.6 mmol L^−1^ (N-NO_3_^−^), 0.4 mmol l^−1^ (P-PO_4_^3−^), 2.1 mmol L^−1^ (K), 0.7 mmol L^−1^ (Mg), 1.4 mmol L^−1^ (Ca) and 0.8 mmol L^−1^ (S-SO_4_^2−^). The micronutrient concentration was: 22.1 µmol L^−1^ (Fe), 40.5 µmol L^−1^ (B), 3.8 µmol L^−1^ (Mn), 2.2 µmol L^−1^ (Zn), 0.2 µmol L^−1^ (Cu) and 0.1 µmol L^−1^ (Mo). Experiments were repeated twice, from 6 November 2020 until 11 December 2020 and from 18 March 2021 until 23 April 2021.

### 4.2. Monitoring Soil Moisture Content 

Irrigation was automated using irrigation thresholds. Commercially available soil moisture sensors (Soil Pro Mini, Sigrow BV, Wageningen, The Netherlands) were used in this research. Substrate volumetric water content was measured every 5 minutes. Per treatment, five soil moisture sensors were inserted in randomly selected containers. As the relation between the volumetric water content of the substrate before irrigation and the irrigation water taken up during irrigation is inversely proportional, the irrigation thresholds were calculated according to the equation *y* = −0.2492*x* + 21.729 (*R*^2^ of 0.81), with *y* the increase in volumetric water content during irrigation (%) and *x* the volumetric water content of the substrate before irrigation (current minimum) (%). When the volumetric water content measured by the sensors went below the calculated irrigation threshold, an irrigation action of 4 minutes was triggered in the photoperiod, between 6:00 a.m. and 10:00 p.m. The applied irrigation thresholds, set-points and actual volumetric water content of the two replications are summarized in Table 5. 

The VWC measured by the sensors (%) was converted to mL container^−1^, an absolute value, using the substrate specific equation *y* = 1.9348*x* + 51.928 (*R*^2^ = 0.93), with *y* the amount of water in substrate (mL container^−1^) and *x* the substrate VWC (%). This linear relation was estimated through linear regression based on 44 data points, for which the substrate water content was calculated from the measured fresh and dry weight of the substrate. Subsequently, this equation was used to determine the evapotranspiration by the plants (mL container^−1^). Daily evapotranspiration (mL day^−1^ container^−1^) was calculated by subtracting the VWC before irrigation (minimum, *n +* 1) from the VWC after irrigation (maximum, *n*). The total evapotranspiration (mL container^−1^) was then calculated through integration of the daily evapotranspiration over the cultivation period. 

### 4.3. 3-Point Bending Tests

At least 50 stems per treatment, from 12 different plant containers, were used to determine the mechanical properties. The stems were cut from the container, placed into plastic bags and transported to the laboratory. Stems similar in size and appearance (Table S1) were recut at the base under water and kept overnight under water with the aerial parts covered with a plastic bag, according to the method described by Jacobsen et al., 2005 [38]. Subsequently, the stems were recut underwater to 50 mm segments and kept underwater for an additional 24 h in 15 mL falcon tubes. Stem segments were placed on the two supporting pins of a 3-point bending setup on a UTM (*Universal Testing Machine,* Ametek LS1 instrument; Lloyd materials testing, Ametek sensors, test & calibration), using a 50 N load cell in such a way that the internodes were always measured. The nodes were excluded from the tests as nodal areas have different anatomical and mechanical characteristics compared to internodes [42,43]. The mechanical properties of basil stems were assessed using a three-point bending test. The stem test section was placed onto the two supporting pins located 30 mm apart, while a third loading pin was lowered from above. Stems were deflected for 5 mm at a constant rate of 0.2 mm s^−1^. The flexural or bending modulus (*E_bend_*) was calculated from the measured force–deformation profile using the following expression, derived from [11,44], for circular beams utilizing the principles of engineering beam-bending theory, as Equation (1):

(1) $$E_{bend}=\frac{L^{3}F_{max}}{4\;\;d^{3}\delta }$$

where:

*E_bend_*—Flexural modulus, bending modulus or modulus of elasticity (MOE) (MPa)
*L*—distance between the two metal supports (mm)
*F_max_*—maximum load or bending force (N)
*d*—diameter of the stem sample (mm)
*δ*—deflection (mm) at maximum force

Equation (1) has been derived for an isotropic material with a uniform cross section, which provides an effective flexural modulus for this study. The flexural or bending modulus is a measure for the stem stiffness, i.e., the stress required to bend the cylinder to a certain strain. Stiff stems will demonstrate a high flexural modulus. As the stress–strain curve for plant stems is not linear, the modulus was evaluated at the maximum force withstood by the stem that allows it to go back to its original dimensions when the load is removed, thus staying within the elastic region. Only the maximum forces (*F_max_*) exerted on the stem samples were analyzed. Similarly, the flexural strength (*σ*) or modulus of rupture (MOR) (MPa) was calculated from the maximal load *F_max_* as Equation (2):

(2) $$\sigma =\frac{8\;F_{max}\;L}{\pi \;d^{3}}$$

As the maximum force (*F_max_*) was used for the calculation of both parameters, both parameters are interrelated. In the first replication of the experiment, the anatomical traits and mechanical stem strength of the apical stem segments, the third internode, were investigated. The anatomical traits and mechanical stem strength of the basal stem segments, the first internode, were researched in the second replication of the experiment. 

### 4.4. Biometric and Anatomical Measurements

At the end of the cultivation period, 37 DAS, macro-morphological traits were measured. Total fresh and dry weight of shoots and leaves were measured separately on 10 containers per treatment. Dry weight was determined by placing the stems in a drying oven for 7 days at 70 °C (ICP500, Memmert GmbH + Co. KG., Büchenbach, Germany). The number of stems per container was counted. 

Twenty stems per treatment were examined for their anatomical traits for each replication. For the first replication, the xylem and collenchyma anatomical measurements were performed on the apical stem segments from similar stems from the same plant containers. For the second replication, adjacent stem segments from the stem test section were used for the xylem and sclerenchyma anatomical measurements on the basal stem segments. Stems were stored in a 30% ethanol/demi-water solution. The stems were cut into sections and viewed under an Olympus BX40 microscope at a 20× magnification. Measurements of xylem vessel diameter (µm) and number, collenchyma, sclerenchyma and tracheid area per cross-sectional stem area (mm^2^) and diameter (µm) were made using the software program ToupView (ToupTek, Hangzhou, China). 

### 4.5. Statistical Analyses 

The Shapiro–Wilk test was used to test the normality of the data. If the assumption of normality was met, an ANOVA followed by a Tukey HSD test was used to compare the means for the different treatments (5% significance level). In the other case, the non-parametric Wilcoxon rank sum test was used to compare the different treatments. All statistical analyses were performed using the JMP Pro 14 (SAS Institute Inc., Cary, NC, USA) program and R Studio (version 4.0.3). The graphical representations of the data were generated using Microsoft Office Excel 2016 and R. Principal component analysis (PCA), summarizing the data from the stem anatomical and mechanical traits at the base of the stem, was conducted with R. 

## 5. Conclusions

The observed morphologies of the xylem vessels and collenchyma indicate that soil moisture content, and by this the water absorption by the plant, has a major impact on the anatomical structure of basil stems. Growth at higher substrate volumetric water content results in both fewer and larger xylem vessels in the stem. Low soil moisture content during the cultivation period stimulates the development of collenchyma, resulting in larger areas of collenchyma. To our knowledge, we are the first to describe this relationship between water availability during the cultivation period and collenchyma development in a quantitative way. 

The difference between the reaction of collenchyma and sclerenchyma, the two crucial elements of mechanical support in higher plants, requires further investigation. The irrigation regime did not have a significant impact on sclerenchyma formation. A possible explanation can be found in the developmental stage of the plants. Collenchyma supports plant organs which undergo extensive turgor-driven elongation, while sclerenchyma with its non-extensible rigid cell walls cannot support growing organs. However, it should be noted that the difference in collenchyma formation between the different treatments cannot have been turgor-driven, as the area fraction of collenchyma increased with decreasing water availability. As the different treatments resulted in similar mean diameters, the increased collenchyma area with decreasing substrate volumetric water content can be attributed to an elevated formation of collenchyma cells. 

Bending resistance is an important feature for plant survival. Depending on the place in the stem, apically or basally, the basil plants were found to use different cells for mechanical support. Collenchyma cells are responsible for resistance to bending in the apical stem segments, while tracheids showed a strong correlation with mechanical stem properties in the basal stem segments, both providing mechanical support to the xylem vessels and overall stem structure. 

Our findings illustrate the relationship between the xylem structural traits associated with water stress and the structural traits associated with mechanical stem strength. Further research is recommended to unravel the underlying mechanisms which regulate collenchyma formation as a function of water availability, because this could pave the way to new approaches to adapt crops to climate change.

## Figures and Tables

**Table 1:** Mean vessel diameter (µm), vessel frequency per mm^2^ and vessel area per total area (%) of the apical and basal stem segments of basil plants cultivated in containers at 37 DAS (days after sowing). All values are means ± 1 SE. *n* refers to the number of vessels over which the parameters were calculated. Different capital letters between brackets indicate significant differences between the treatments for the given parameter, according to a Tukey test (*p* ≤ 0.05).

Treatment	*n*	Mean Vessel Diameter (µm)	Vessel Frequency per mm^2^	Percent Vessel Area per Transverse Stem area (%)
**Apical stem segment**				
pF1.95	794	15.42 ± 1.62 (B)	83 ± 17 (A)	1.25 ± 0.20 (A)
pF1.65	625	20.51 ± 1.68 (A)	49 ± 7 (B)	1.39 ± 0.16 (A)
pF1.30	551	19.13 ± 1.98 (A)	55 ± 11 (B)	1.29 ± 0.22 (A)
pF1.15	523	19.93 ± 3.25 (A)	48 ± 12 (B)	1.11 ± 0.17 (A)
**Basal stem segment**				
pF1.95	3534	25.29 ± 2.39 (B)	40 ± 5 (A)	1.99 ± 0.43 (C)
pF1.65	3573	33.65 ± 2.60 (A)	29 ± 4 (B)	2.57 ± 0.43 (A)
pF1.30	3129	34.25 ± 3.20 (A)	24 ± 3 (C)	2.19 ± 0.33 (BC)
pF1.15	3482	34.73 ± 3.51 (A)	26 ± 6 (BC)	2.41 ± 0.46 (AB)

**Table 2:** Mean comparison (±SD) of the flexural modulus and flexural strength of basil stems cultivated at different soil moisture contents for the apical stem segments. Different letters within columns indicate significant differences between the treatments, according to a Wilcoxon test with *p* ≤ 0.05.

Treatment	*n*	Flexural Modulus *E_bend_* (MPa)	Flexural Strength *σ* (MPa)
pF1.95	60	48.08 ± 43.07 (a)	0.36 ± 0.23 (a)
pF1.65	56	45.14 ± 39.04 (a)	0.34 ± 0.19 (a)
pF1.30	53	40.67 ± 39.10 (ab)	0.29 ± 0.21 (ab)
pF1.15	56	26.82 ± 24.59 (b)	0.27 ± 0.21 (b)

**Table 3:** Mean (± SD) tracheid and sclerenchyma areas per stem area (%) and mean tracheid and sclerenchyma diameters (µm) of the basal stem segments for the four different irrigation treatments (*n* = 20). Different letters within columns indicate significant differences (*p* < 0.05) between the treatments, according to the Wilcoxon test (tracheid area) and Tukey test (sclerenchyma, mean tracheid and sclerenchyma diameter).

Treatment	*n*	Tracheid Area per Stem Area (%)	Mean Tracheid Diameter (µm)	Sclerenchyma per Stem Area (%)	Mean Sclerenchyma Diameter (µm)
pF1.95	20	18.14 ± 7.60 (a)	10.53 ± 3.46 (a)	3.54 ± 0.81 (a)	20.95 ± 5.87 (a)
pF1.65	20	18.31 ± 8.78 (a)	10.89 ± 3.83 (a)	3.33 ± 0.85 (a)	20.20 ± 6.06 (a)
pF1.30	20	15.94 ± 5.69 (a)	11.02 ± 3.49 (a)	3.29 ± 0.46 (a)	22.62 ± 6.65 (a)
pF1.15	20	19.56 ± 9.54 (a)	10.81 ± 3.44 (a)	3.46 ± 0.44 (a)	22.64 ± 6.80 (a)

**Table 4:** Correlations between the different treatments, stem anatomical traits and mechanical characteristics. Strength of correlations (r^2^) between the different parameters on a significance level of *p* < 0.01 are shown.

Parameters	Tracheid Area per Stem Area (%)	Sclerenchyma per Stem Area (%)	Vessel Frequency per mm^2^	Vessel Diameter (µm)	Percent Vessel Area Per Stem Area (%)
VWC (%)	0.03	−0.05	−0.68	0.67	0.21
Flexural modulus *E_bend_* (MPa)	0.76	0.06	0.18	−0.15	0.00
Flexural strength *σ* (MPa)	0.86	0.10	0.21	−0.04	0.27

**Table 5:** Comparison of the irrigation set-points and their corresponding irrigation thresholds with the actual average volumetric water content ± SD for the 5-week cultivation, for replication 1 and replication 2, measured by the soil moisture sensors.

Irrigation Set-Point (%)	Calculated Irrigation Thresholds (%)	Actual Average Volumetric Water Content of the Substrate during the 5-Week Experiment (%) 1st Replication	Actual Average Volumetric Water Content of the Substrate during the 5-Week Experiment (%) 2nd Replication
20	11	26 ± 4	21 ± 9
30	22	31 ± 5	32 ± 8
40	34	44 ± 9	39 ± 13
50	45	49 ± 8	51 ± 8

## Supplementary Materials

The following are available online at https://www.mdpi.com/article/10.3390/plants10071320/s1, Table S1: Mean (±SD) stem diameters of the apical and basal stem segments for the four different irrigation treatments (*n* = 20). Different letters within columns indicate significant differences between the treatments according to the Tukey test (*p* ≤ 0.05).
