# Supplementary material for: Soil Moisture Levels Affect the Anatomy and Mechanical Properties of Basil Stems (Ocimum basilicum L.)
Source: Plants (Basel). 2021 Jun 28;10(7):1320. doi: 10.3390/plants10071320 (PMC8309113; doi:10.3390/plants10071320)
Supplement: Supplementary file 1 [file plants-10-01320-s001.zip › plants-1258617-supplementary.pdf]

Supplementary Materials:

Table S1. Mean (±SD) stem diameters of the apical and basal stem segments for the four different irrigation treatments (n=20). Different letters within columns indicate significant differences between the treatments according to the Tukey test ($p \leq 0.05$).

Treatment	Stem Diameter Apical Stem Segments (mm)	Stem Diameter Basal Stem Segments (mm)
pF1.95	1.85 ± 0.64 (a)	2.26 ± 0.34 (a)
pF1.65	1.78 ± 0.54 (a)	2.53 ± 0.49 (a)
pF1.30	2.01 ± 0.73 (a)	2.63 ± 0.43 (a)
pF1.15	2.08 ± 0.74 (a)	2.54 ± 0.55 (a)
